# Knowledge mapping of students’ mental health status in the COVID-19 pandemic: A bibliometric study

**DOI:** 10.3389/fpsyg.2022.985866

**Published:** 2022-11-23

**Authors:** Yang Yang, Qingtai Cao, Mingyi Zhao, Quan Zhuang

**Affiliations:** ^1^Department of Pediatrics, The 3rd Xiangya Hospital, Central South University, Changsha, Hunan, China; ^2^Transplantation Center, The 3rd Xiangya Hospital, Central South University, Changsha, Hunan, China; ^3^Department of Educational Affairs, The 3rd Xiangya Hospital, Central South University, Changsha, Hunan, China; ^4^Research Center on Transplantation Medicine of National Health Ministry, Changsha, Hunan, China

**Keywords:** COVID-19, student, mental health, psychology stress, bibliometrics

## Abstract

**Objective:**

The purpose of this study was to investigate the international scientific output on mental health of students during COVID-19 from 2020 to 2022 through a bibliometric analysis and to explore trend and research hotspots in this field.

**Methods:**

We searched the Web of Science Core Collection for publications and used a variety of software to analyze and visualize the data such as R, CiteSpace, VOSviewer and Scimago.

**Results:**

A total of 2,734 publications were retrieved as of June 4, 2022, published by 3,894 institutions from 120 countries/regions. China and the United States lead in the quantity and quality of publications in this field. According to Bradford’s Law, 16 journals are considered core journals in the field. Co-cited references indicate the main psychological problems of students under the epidemic revolve around anxiety, poor sleep and financial difficulty. Their behavior might also be influenced by increased internet and alcohol use.

**Conclusion:**

Mental health of students during COVID-19 is attracting increasing attention. It is identified that the research hotspots in this field continue to revolve around emotional anxiety and unhealthy behaviors. Due to the different troubles faced by different groups under COVID-19, further exploration of the relevant factors specific for students are needed, with a hopeful view to providing ideas for intervention measures.

## Introduction

The coronavirus disease 2019 (COVID-19) is an emerging epidemic caused by severe acute respiratory syndrome coronavirus 2 (SARS-CoV-2; Wang D. et al., 2020; Hu et al., 2021). Since 2019, the rapidly spreading COVID-19 pandemic has caused a global health threat (Fang et al., 2022). It is important to note that the COVID-19 pandemic is not only taking a toll on people’s physical health, but also has a negative impact on people’s mental health (Zhang and Chen, 2021; Pappa et al., 2022; Zhang et al., 2022).

As a vulnerable group, students are relatively more prone to negative psychological symptoms (Cao et al., 2020; Copeland et al., 2021). Numerous studies have shown that during the COVID-19 pandemic, students’ mental health has been severely challenged, regardless of their academic stage. A cross-sectional study using data from the Mental Health Survey of School-aged Children and Adolescents in Guangdong Province, China, showed that the prevalence of self-reported psychological distress among students was relatively high during the COVID-19 pandemic (Qin et al., 2021). A large-scale survey of 746,217 college students revealed that acute stress, anxiety, and depressive symptoms are prevalent during the COVID-19 pandemic, and that multiple epidemiological and psychosocial factors are associated with an increased risk of mental health problems (Ma et al., 2020). The mental health of master’s, doctoral and postdoctoral researchers has also been severely negatively impacted by the pandemic (Salim, 2021). This study aims to explore mental health-related conditions of students during the COVID-19 outbreak. We hypothesize that students’ mental health has been widely challenged during the COVID-19 pandemic and needs more care and support.

Over the past 2 years, a raft of research has been published on student mental health during the COVID-19 pandemic. However, there is no literature that systematically evaluates the relevant published literature. Bibliometrics uses mathematical and statistical methods to quantitatively analyze a large amount of literature in a specific research field, exploring research aspects and research trends in that field (Contreras-Barraza et al., 2021; Shen et al., 2022). Many researchers have used bibliometrics to evaluate their fields of study (Contreras-Barraza et al., 2021; Darroudi et al., 2021; Peng et al., 2022). However, no specific bibliometric studies have been conducted to date on the knowledge graph of student mental health during the COVID-19 pandemic. We have used bibliometrics to understand what is the current state of research work in this field, and question what are the research priorities and new trends? And further find out new problems that should addressed and possible directions to solve the problem. Therefore, we used R software, Scimago software, and citespace software to evaluate the literature on psychological stress of students during the COVID-19 period up to June 4, 2022 by bibliometric method, and generated knowledge maps. The results of these analyses can describe the current state of the field and identify new research directions.

## Materials and methods

### Literature retrieval

The literature retrieval was performed on June 4, 2022, using Web of Science Core Collection (WoSCC) to search for publications on research on psychological stress among students during COVID-19. The search terms we used were as follows: Topic: (COVID-19 OR SARS-CoV-2 OR 2019-nCov) and (mental health OR psychological stress) and (student OR pupil OR undergraduate OR freshman OR sophomore OR junior OR senior OR graduate OR master OR doctoral student). Among them articles and review articles were included in the study. All the records retrieved from the WoSCC were downloaded independently by two authors (QC and YY). A total of 2,734 documents published from 2020 to 2022 met the search criteria for this study.

### Data collection and analysis

We exported a complete data record from WoSCC, including the number of annual publications, countries, journals, citations, impact factor (IF) and Hirsch index (H-index), etc. Firstly, the top 10 disciplines with annual publications were chosen to investigate the major study categories in this field. Accumulative publication, average citations per publication and H-index of top 10 countries with the most publications were analyzed to evaluate the scientific impact of the countries/regions. R version 4.0, a free software environment for statistical computing and graphics, was used to show the above results.

Cooperative networks between countries/regions and institutions were visualized by Scimage software. Co-cited author analysis was conducted, which was further clustered by CiteSpace. CiteSpace V software was used to conduct co-citation analysis of the references and further to group the references into 16 clusters. Next, a timeline view of co-cited references was constructed. The journals were also clustered by CiteSpace algorithm and the core journals were selected according to Bradford’s Law. CiteSpace could capture keywords with strong citation bursts and construct visualization maps of all items. A citation burst is a key indicator for identifying emerging trends (Chen et al., 2020). We used double image overlay to emphasize the key words of 2022 in CiteSpace. Conceptual analysis was performed using the R package “Bibliometrix”.^1^

## Results

### The overall trend of publications from 2020 to 2022

**Figure 1:**
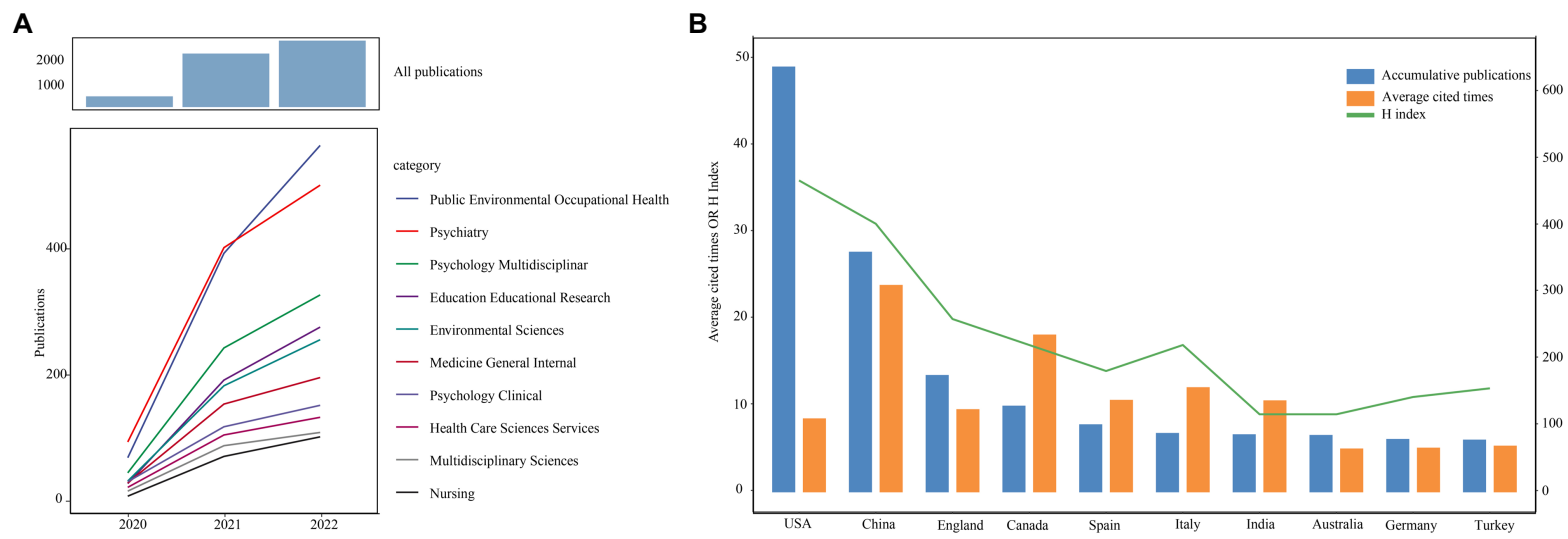
The overall distribution of publications. **(A)** The annual accumulative publications were exhibited by year and discipline. **(B)** The total number, average cited time and H-index of publications in countries.

Information visualization was used to analyze articles on psychological stress among students during COVID-19 from WoSCC database from Jan 1, 2020 to June 4, 2022. The total number of cumulative publications in 2020, 2021, and 2022 was 439, 2,202, and 2,734, respectively (Figure 1A). The number of publications in 2021 was around four times than that of 2020, indicating that more attention in this field was obtained after the COVID-19 outbreak and high-quality articles erupted in 2020. Next, the co-cited reference analysis was carried out to explore which articles distinctively contributed to the development of this area. The most 10 co-cited articles were all published in 2020, the first three of which were authored by Cao WJ, Brooks SK and Wang CY, respectively (Table 1).

**Table 1:** Top 10 co-cited references in mental health of students in COVID-19.

Rank	Count	Year	Author	Title
1	494	2020	Cao WJ	The psychological impact of the COVID-19 epidemic on college students in China
2	348	2020	Brooks SK	The psychological impact of quarantine and how to reduce it: rapid review of the evidence
3	288	2020	Wang CY	Immediate Psychological Responses and Associated Factors during the Initial Stage of the 2019 Coronavirus Disease (COVID-19) Epidemic among the General Population in China
4	193	2020	Son C	Effects of COVID-19 on College Students’ Mental Health in the United States: Interview Survey Study
5	161	2020	Xiong JQ	Impact of COVID-19 pandemic on mental health in the general population: A systematic review
6	151	2020	Qiu JY	A nationwide survey of psychological distress among Chinese people in the COVID-19 epidemic: implications and policy recommendations
7	147	2020	Holmes EA	Multidisciplinary research priorities for the COVID-19 pandemic: a call for action for mental health science
8	147	2020	Sahu P	Closure of Universities Due to Coronavirus Disease 2019 (COVID-19): Impact on Education and Mental Health of Students and Academic Staff
9	146	2020	Odriozola-Gonzalez P	Psychological effects of the COVID-19 outbreak and lockdown among students and workers of a Spanish university
10	145	2020	Wang CY	A longitudinal study on the mental health of general population during the COVID-19 epidemic in China

The disciplines of this area were mainly about “psychology,” “environment” and “medicine.” The disciplines with the most publications are “public environmental occupational health” and “psychiatry,” the annual publication trend of which were consistent to the total publications (Figure 1A).

### Country/region publication and collaboration analysis

All publications in the field were distributed among 3,894 institutions from 120 countries/regions, of which the production of the United States ranked the first with 749 documents by far, followed by China (445), the United Kingdom (216), Canada (156) and India (120) (Figure 1B, Table 2). The whole trend of H index was consistent with that of all publications where the United States (40), China (33) and the United Kingdom (21) ranked the first three. With reference to the average citations per articles, different from H index, China (20.82) and Canada (18.28) showed the highest average cited times while the United States (8.6) was relatively lower. Overall, the United States and China are the more enthusiastic and leading in research in this field.

**Table 2:** The top 10 productive countries/regions related to mental health in students under COVID-19.

Rank	Countries/Region	Publication	Average per item	H-Index
1	the United States	749	8.6	40
2	China	445	20.82	33
3	The United Kingdom	216	9.09	21
4	Canada	156	18.28	17
5	India	120	8.02	9
6	Italy	119	12.04	19
7	Spain	119	10.5	15
8	Australia	114	9.53	13
9	Turkey	104	4.76	13
10	Germany	98	5.15	11

**Figure 2:**
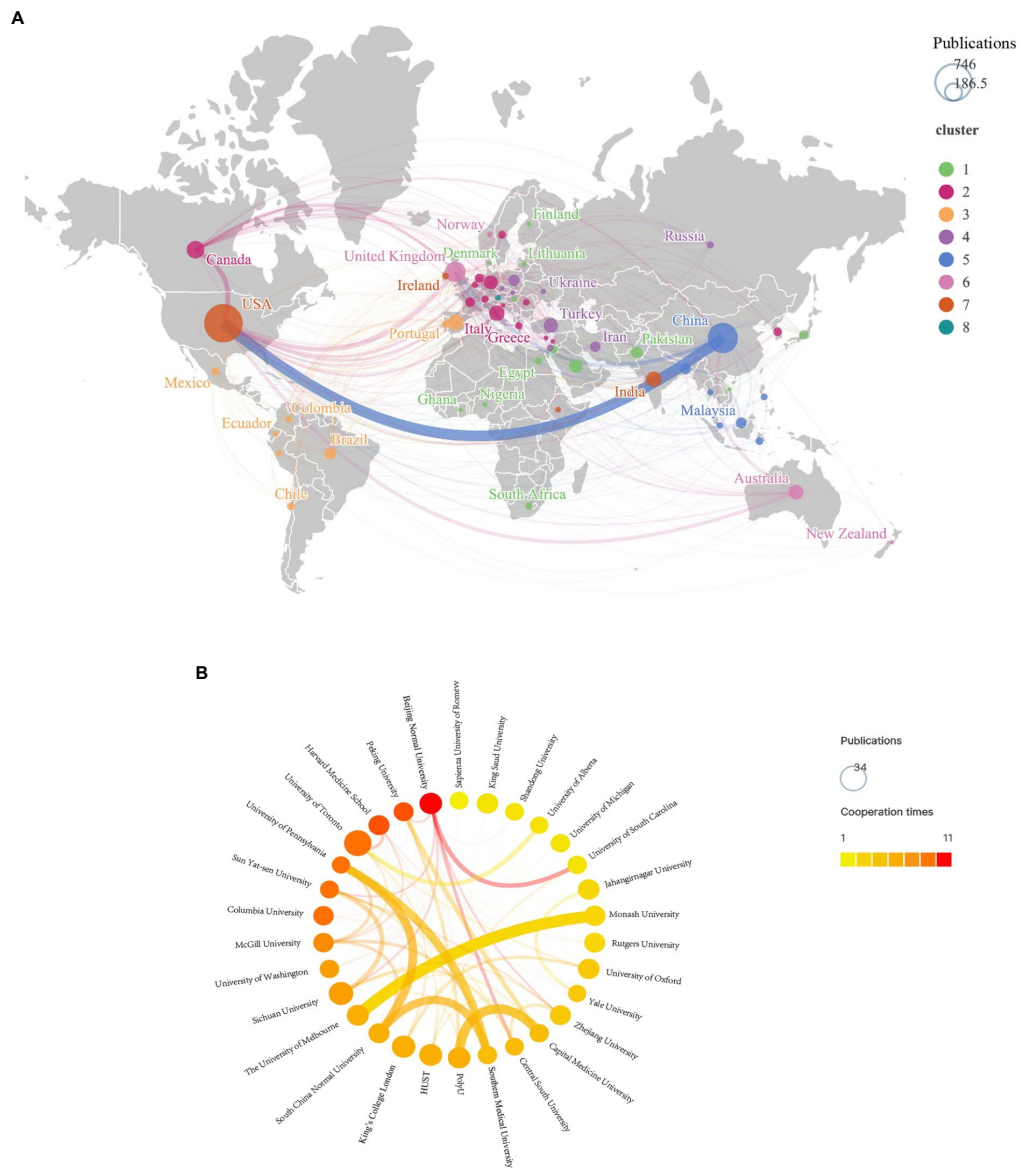
Collaboration visualization between countries/regions or institutions. **(A)** Collaboration analysis of countries/regions. **(B)** Collaboration analysis of instructions.

Next, the inter-country collaborations and inter-agency partnership were investigated. Figure 2A presents the analysis of cooperation between countries. The countries/regions that cooperated closely were clustered together and given the same color. The thicker the line, the closer the cooperation between the two countries. The results showed that China and the United States had the closest cooperation in this area. The collaboration among the United States, Canada and the United Kingdom were also relatively closer, consistent to the publication trend. A network diagram showed partnerships between major institutions (Figure 2B). Circles represent institutions, and lines represent partnerships. The deeper the circle, the more papers the institution published in the field, and the thicker the line, the closer the cooperation between the two institutions. The strongest collaboration relationship was found between The University of Melbourne and Monash University.

### Co-cited author analysis

**Figure 3:**
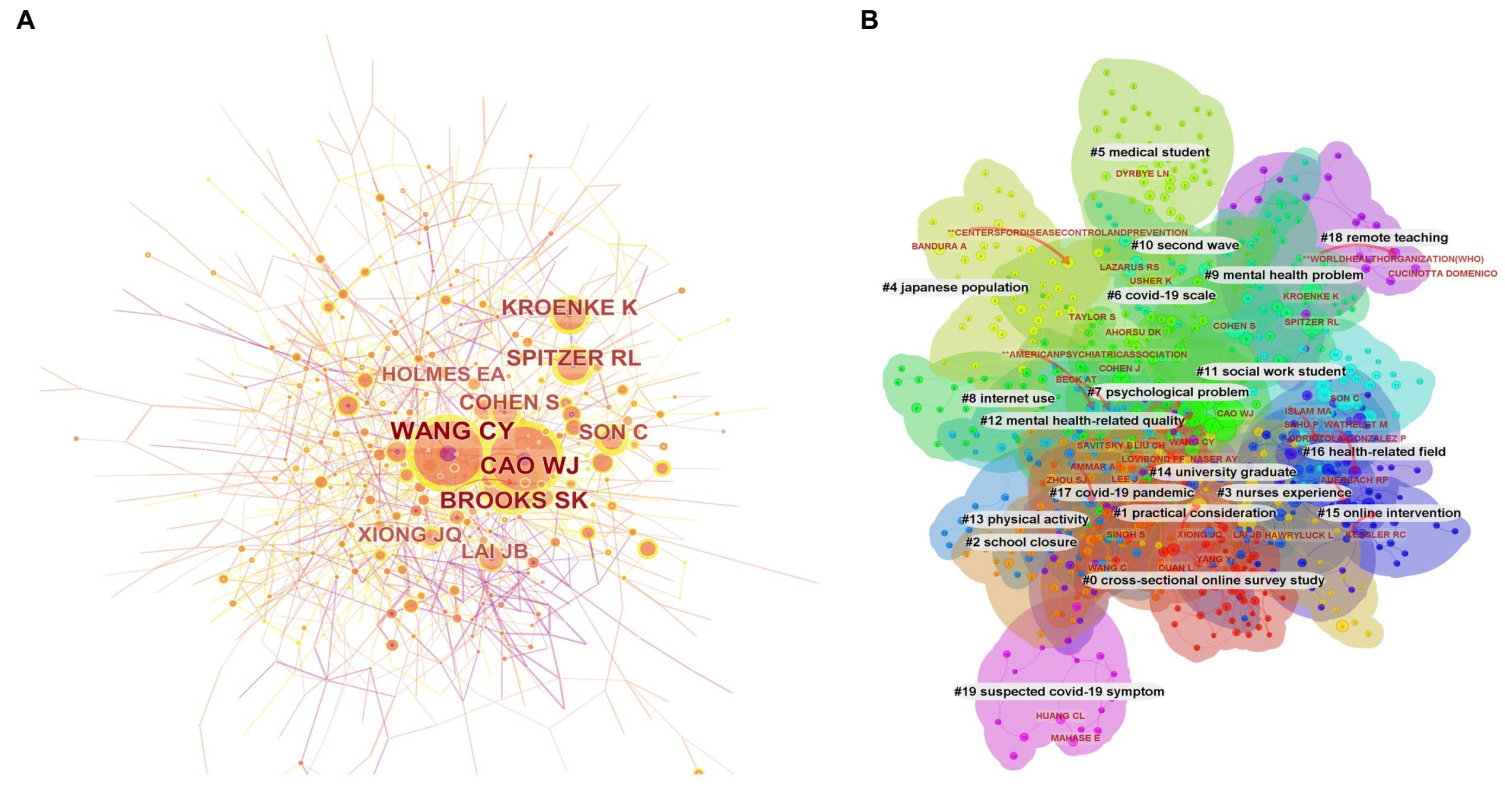
CiteSpace network visualization map of co-cited authors and their cluster results. **(A)** The co-cited author analysis. **(B)** Clusters of co-cited authors.

The selected 2,734 publications were produced by 12,889 authors and the top 10 productive authors and co-cited authors were listed in Table 3. Mark D Griffiths headed with 10 documents, followed closely by Teris Cheung, Yutao Xiang and Jingbo Zhao who all published 9 articles. The most frequently co-cited author was Wang CY with 565 co-cited times, followed by Cao WJ (550) and Brooks SK (455). These three authors were also the authors who wrote the most co-cited articles in this field (Table 1), revealing the significant effect of them. The author’s co-cited network analysis was visualized in Figure 3. Subsequently, we performed a cluster analysis of co-cited authors (Figure 3B). Most authors were clustered into “#0 Cross-sectional online survey study,” indicating the research in this field mainly focused on online questionnaires and cross-sectional surveys.

**Table 3:** Top 10 productive authors and co-cited authors related to ferroptosis.

Citing authors			Cited authors		
Rank	Author	Count	Rank	Co-cited author	Citation
1	Mark D Griffiths	10	1	Wang CY	565
2	Teris Cheung	9	2	Cao WJ	550
3	Yutao Xiang	9	3	Brooks SK	455
4	Jingbo Zhao	9	4	Spitzer RL	295
5	Mohammad Nurunnabi	8	5	Kroenke K	283
6	Kamilah Kamaludin	7	6	Son C	243
7	Heba Bakr Rhoshaim	7	7	Cohen S	221
8	Caruthan Chinna	7	8	Lai JB	190
9	Cezary Kusnierz	6	9	Xiong JQ	178
10	Fangbiao Tao	6	10	Holmes EA	171

### Reference co-citation analysis

**Figure 4:**
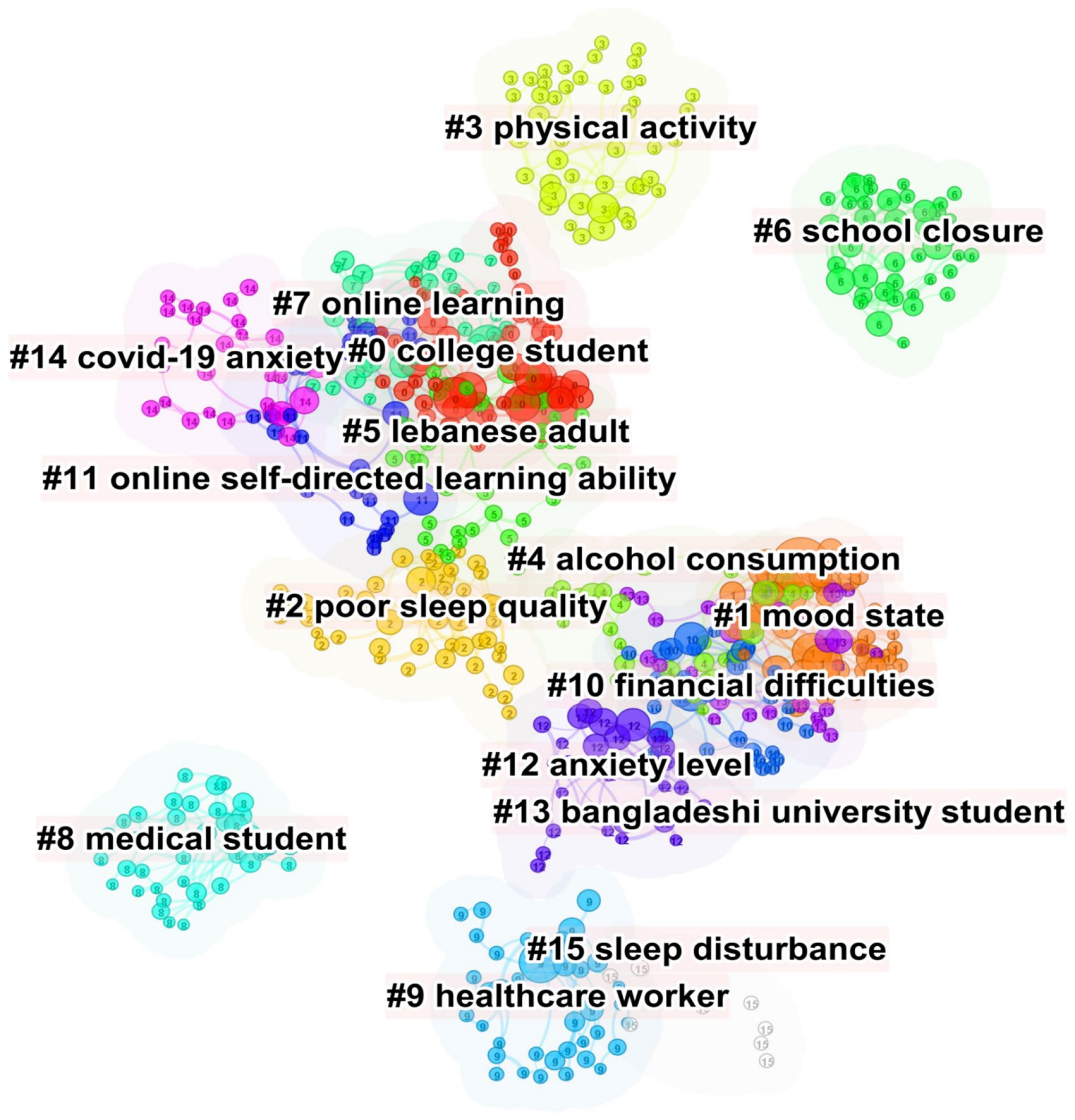
Cluster analysis of co-cited references.

A total of 41,321 co-cited references were visualized by CiteSpace with time slice set as a year (Figure 4). The references were clustered according to the log-likelihood ratio (LLR) algorithm, and 16 clusters were obtained (Figure 4, Table 4). The largest cluster is “college student” (cluster #0), followed by “mood state” (cluster #1), “poor sleep quality” (cluster #2), and “physical activity” (cluster #3). It is not surprising that college students were the most frequently studied group as it’s easier to collect their questionnaires. We further explored the cluster 1 and cluster 2, which both reflected the mental situation of students facing COVID-19. Tang WJ (89), Kaparounaki CK (80), Liu CH (62), Lei L (51) and Cellini N (39) were the most cited authors in “mood state,” and Husky MM (99), Auerbach RP (67), Zhang Y (66), Ammar A (45) and Stanton R (40) were the most cited authors in “poor sleep quality.” It was highly recommended to read these authors’ documents if related research needed to be conducted. Besides, “cluster 4: alcohol consumption,” “cluster 7: online learning” and “cluster 8: medical students” were of interest and the related authors were shown in Supplementary Table 1.

**Table 4:** Major clusters of co-cited references.

Cluster ID	Size	Silhouette	Year Ave.	Label (LLR)
0	51	0.922	2019	College student
1	49	0.839	2019	Mood state
2	49	0.771	2020	Poor sleep quality
3	48	0.858	2019	Physical activity
4	44	0.868	2019	Alcohol consumption
5	43	0.797	2019	Lebanese adult
6	42	0.952	2019	School closure
7	41	0.815	2019	Online learning
8	41	0.908	2019	Medical student
9	38	0.862	2019	Healthcare worker
10	34	0.796	2019	Financial difficulties
11	32	0.875	2019	Online self-directed learning ability
12	31	0.874	2019	Anxiety level
13	31	0.847	2019	Bangladeshi university student
14	28	0.805	2019	COVID-19 anxiety
15	9	0.989	2019	Sleep disturbance

Clusters are referred in terms of the labels selected by log-likelihood ratio test method (LLR).

**Figure 5:**
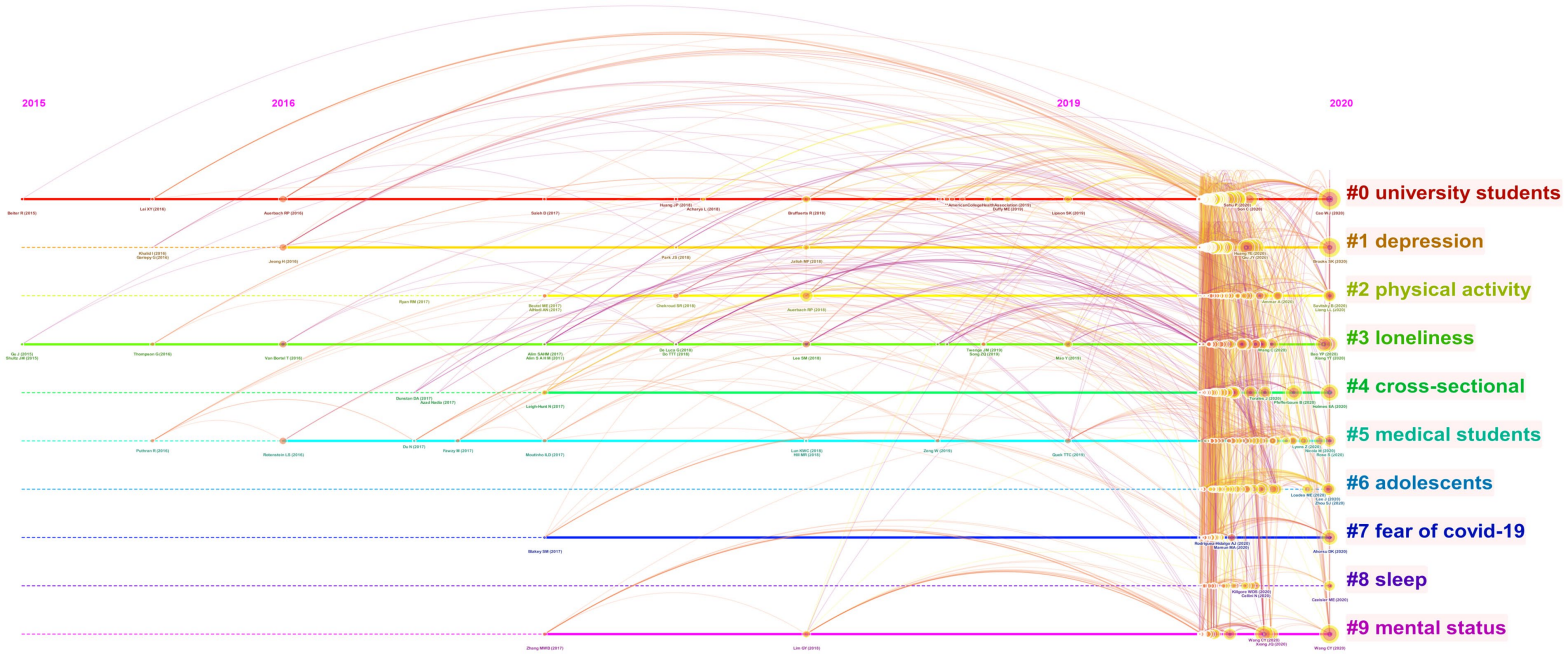
Timeline view of co-cited references related to mental health of students during COVID-19.

Afterwards, we constructed a timeline view of co-cited references to illustrate topic distribution in the field, and also investigated topic trends and interrelationships over time (Figure 5). In the timeline view, we found a sharp increase in the number of documents per cluster since 2019. It is apparent that this resulted from the outbreak of COVID-19.

### Journal analysis

In total, 927 journals have contributed to the 2,734 publications. According to Bradford’s Law, 16 journals were considered core journals in the field (Table 5), among which INT J ENV RES PUB HE contained the largest number of publications (219 publications), followed by FRONT PSYCHOL (158 publications) and FRONT PSYCHIATRY (115 publications). Concerning Impact Factor (IF), INT J MENT HEALTH AD has the highest IF (IF = 11.555) in 2019, followed by PSYCHIAT RES (IF = 11.225) and J AFFECT DISORDERS (IF = 6.533). In the quartile category, 5 of the 16 journals were in Q1 (the top 25% of the IF distribution) of different areas, 9 of 16 were in Q2 and the rest were in Q3.

**Table 5:** Core journals related to the research of mental health of students during COVID-19.

Citing journals					Cited journals				
Rank	Journal	Publications	IF	Quartile	Rank	Journal	Co-cited times	IF	Quartile
1	INT J ENV RES PUB HE	219	4.614	Q2	1	INT J ENV RES PUB HE	1,378	4.614	Q2
2	FRONT PSYCHOL	158	4.232	Q1	2	PSYCHIAT RES	1,302	11.225	Q1
3	FRONT PSYCHIATRY	115	5.435	Q2	3	PLOS ONE	1,136	3.752	Q2
4	J AM COLL HEALTH	58	2.395	Q3	4	J AFFECT DISORDERS	980	6.533	Q1
5	PLOS ONE	50	3.752	Q2	5	LANCET	973	202.731	Q1
6	FRONT PUBLIC HEALTH	48	6.461	Q1	6	FRONT PSYCHOL	861	4.232	Q1
7	J AFFECT DISORDERS	36	6.533	Q1	7	LANCET PSYCHIAT	712	7.35	Q1
8	CURR PSYCHOL	32	2.387	Q3	8	FRONT PSYCHIATRY	623	5.435	Q2
9	BMJ OPEN	29	3.017	Q2	9	J MED INTERNET RES	592	7.093	Q1
10	HEALTHCARE-BASEL	28	0.92	Q2	10	BRAIN BEHAV IMMUN	554	19.227	Q1
11	SUSTAINABILITY-BASEL	28	3.889	Q2	11	PSYCHOL MED	511	10.592	Q1
12	INT J MENT HEALTH AD	27	11.555	Q1	12	ASIAN J PSYCHIATR	495	13.89	Q1
13	BMC PSYCHIATRY	25	4.144	Q2	13	JAMA-J AM MED ASSOC	486	157.335	Q1
14	BMC MED EDUC	22	3.263	Q2	14	BMC PUBLIC HEALTH	456	4.135	Q2
15	HELIYON	22	3.776	Q2	15	JAMA NETW OPEN	424	13.366	Q1
16	PSYCHIAT RES	21	11.225	Q1	16	BMJ-BRIT MED J	408	93.467	Q1

The most co-cited journal was also INT J ENV RES PUB HE (1,378 citations), followed by PSYCHIAT RES (1,302 citations) and PLOS ONE (1,136 citations). 12 of the 16 co-cited journals were in Q1 and the rest were in Q2. These results showed that INT J ENV RES PUB HE, FRONT PSYCHOL and PLOS ONE had significant contributions in this field due to the higher publications and citations.

### Keyword analysis

**Figure 6:**
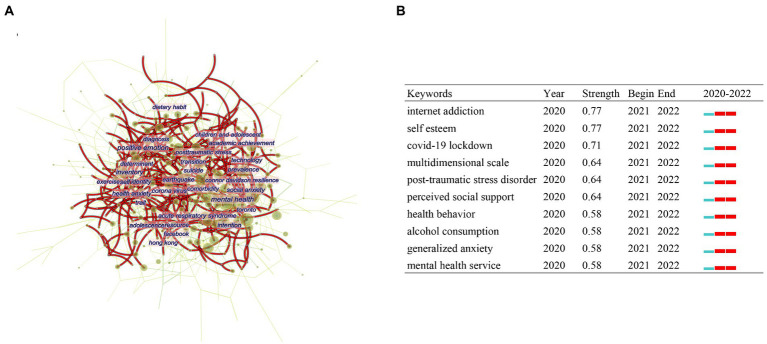
Keyword analysis of the research on mental health of students during COVID-19. **(A)** CiteSpace network visualization map of co-occurring keywords. **(B)** Top 10 burst keywords.

Citespace was used for keyword contribution analysis and keyword burst analysis from 2019 to 2022. Figure 6A shows the keyword contribution map from 2020 to 2022. Dual image overlay was used to investigate the keyword change trend in this field. The red line in Figure 6A represents the keyword contribution since 2022. No obvious change, however, was found. The main keywords were still about “mental health,” “anxiety,” and “stress.” Top 10 keywords with the strongest citation bursts were detected to provide helpful insights to research hotspots in this field (Figure 6B). All the bursts occurred in 2021 and lasted to 2022, some of which were overlapped with the clusters of co-cited references. Under the circumstance of COVID-19, the mental health issues post-traumatic stress disorder, anxiety and self-esteem might influence students. It was worth investigating how COVID-19 influenced students’ behavior on internet and alcohol use.

### Conceptual structure analysis

**Figure 7:**
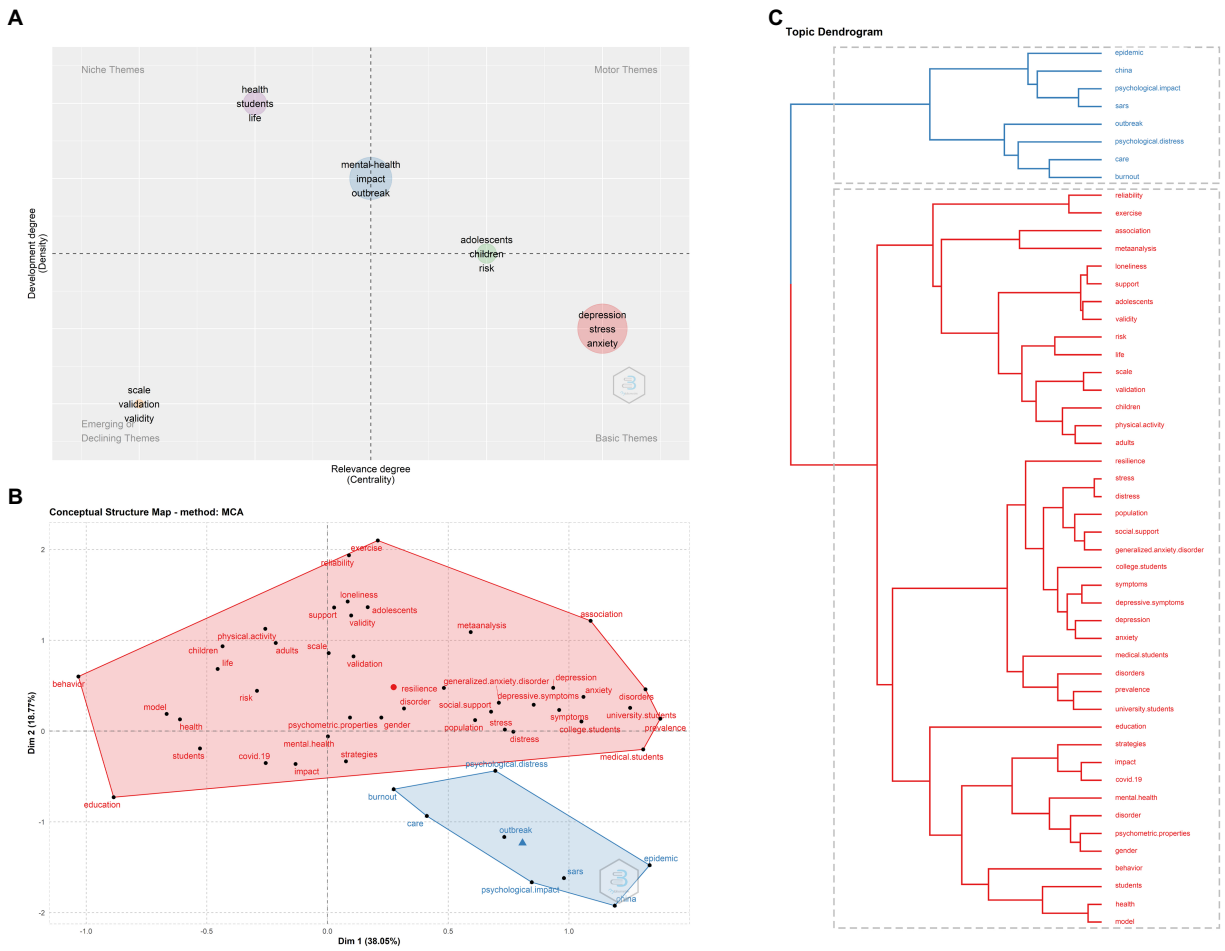
Conceptual analysis of mental health of students during COVID-19. **(A)** Thematic map of related research. **(B)** Word map of factorial analysis. **(C)** Topic dendrogram of factorial.

The thematic map developed from the keywords was shown in Figure 7A, to examine the thematic concepts in the research field of students’ mental health during COVID-19, based on the typology of topics. No themes were found in the upper right quadrant. The basic themes in the lower right quadrant included “depression,” “stress” and “anxiety,” indicating the high centrality but low development degree. The upper left quadrant contained “health,” “students” and “life” with high development but low centrality, which was considered as niche themes. Themes such as “scale,” “validation” and “validity” were emerging or declining themes in the lower left quadrant, meaning the low centrality and low development degree. Moreover, there were also subjects located in the boundary between two quadrants. “Mental-health,” “impact” and “outbreak” were in the boundary between motor themes and niche themes, which had middle centrality and high development degree. Also, “adolescents,” “children” and “risk” were motor themes and basic themes simultaneously with high centrality and middle development degree.

Multiple correspondence analysis (MCA) was used to construct a conceptual structure of this field. All keywords were classified into two clusters in blue and red (Figure 7B). Connection between the two clusters was shown in Figure 7C. The words in blue were mainly associated with COVID-19 and its influence such as “outbreak,” “epidemic” and “psychological impact.” The red cluster represented words related to the influenced population and specific emotions including “students,” “adolescents,” “university students,” “anxiety” and “depression.”

## Discussion

Overall, this paper aims to investigate the output of international scientific publications on student mental health during the COVID-19 pandemic from 2020 to 2022 through a bibliometric analysis, and to explore research trends and hotspots in the field. We found that China and the United States lead in the quantity and quality of publications in this field. Importantly, 16 journals are considered core journals in the field according to Bradford’s Law. In addition, the co-cited references indicated that the main psychological problems of students under the epidemic were closely related to anxiety, poor sleep and financial difficulties. Their behavior may also be affected, such as increased internet usage and alcohol consumption.

In 2020, with the rapid spread of the COVID-19 pandemic, more and more countries have entered partial or nationwide lockdowns (To et al., 2021). Under this circumstance, the mental health of students during the COVID-19 pandemic received greater attention, and a total of 439 relevant publications were published. Between 2021 and 2022, the number of publications in this field increased dramatically. The cumulative number of related articles published in 2021 was 2,202, and in 2022, the number has risen to 2,734. This dramatic increase may be related to the growing physical, psychological, and social challenges that students faced during the pandemic. In terms of the number of relevant publications, the United States and China have the most publications in this field; China and Canada have the highest average citations; the United States and China have the highest H-index. From this perspective, China and the United States are leading the way in this research area. In addition, country/regional cooperation analysis also indicates that China and the United States have established close cooperation in this field. In the institutional collaboration analysis, the University of Toronto and Sichuan University have the most publications. In addition, Beijing Normal University, Peking University, and Harvard Medical School have the most cooperation occurrences, which shows that they are very active in inter-institutional collaborations in this field.

In the analysis of co-cited authors, Wang CY, Cao WJ, Brooks SK, Spitzer RL, Kroenke K, Son C, Cohen S, Lai JB, Xiong JQ and Holmes EA contributed the top 10 co-citations in turn. These researchers have made considerable contributions in this field.

According to the major clusters of co-cited references, we found that the hotspots of related research mainly focus on emotional status (mood state, anxiety level, COVID-19 anxiety), living habits (physical activity, alcohol consumption), learning status (school closure, online learning, online self-directed learning ability), sleep status (poor sleep quality, sleep disturbance), economic pressure (financial difficulties), and medical students (medical student, healthcare worker).

In Table 3, the most co-cited reference is “The psychological impact of the COVID-19 epidemic on college students in China” published by Cao WJ in 2020, and the co-citation count is 547. They conducted a sample analysis of college students at Changzhi Medical College and found that economic impact, daily life impact, and delay in academic activities were positively correlated with anxiety symptoms, while social support was negatively correlated with anxiety levels (Cao et al., 2020). At the same time, they made a recommendation for monitoring the mental health of college students during the COVID-19 pandemic. The second most co-cited reference was the review “The psychological impact of quarantine and how to reduce it: rapid review of the evidence.” Its authors, Brooks SK et al. published it in The Lancet in 2020. They examined the psychological effects of quarantine using three electronic databases. They examined the psychological effects of isolation using three electronic databases, most of which reported negative psychological effects (Brooks et al., 2020). Following these two papers is the article “Immediate Psychological Responses and Associated Factors during the Initial Stage of the 2019 Coronavirus Disease (COVID-19) Epidemic among the General Population in China” by Wang et al. Their findings suggest that females, students and people with specific physical symptoms are more likely to be associated with greater psychological impact and higher levels of stress, anxiety and depression (Wang C. et al., 2020). It is worth mentioning that the three authors, Cao WJ, Brooks SK and Wang CY, are also among the top three authors in the co-citation analysis of authors.

Research on student mental health during the COVID-19 pandemic has gained welcome interest from many journal editors. Among them, the 10 journals in Table 4 contributed the most. The top journals by number of publications are INT J ENV RES PUB HE, FRONT PSYCHOL, and FRONT PSYCHIATRY.

We performed keyword analysis and burst keyword analysis using CiteSpace. In the Keyword Contribution Graph, many keywords have made a large contribution, which shows that the research focus in this field is very diverse. In burst keyword analysis, the top 10 burst keywords in Figure 6 all appeared in 2021, and have maintained a high research interest in 2022. The keywords with the highest burst strength are internet addiction and self-esteem. Through a latent class analysis of Chinese schoolchildren, I-Hua Chen et al. found that during the recovery period of the COVID-19 outbreak, the two groups with a higher level of problematic internet use (PIU) had significantly higher levels of fear of COVID-19 than the one with a lower level (Chen et al., 2021). In addition, a study of residential college students during the COVID-19 lockdown found that the prevalence of PIU was high among residential college students during the COVID-19 lockdown, suggesting that the lockdown policy inevitably had an impact on their social lives. During such stressful events, they are vulnerable to PIU (Xia et al., 2021). Not only in China, many countries have reported the problem of internet addiction among students during the COVID-19 pandemic (Ismail et al., 2021; Kim et al., 2021; Shehata and Abdeldaim, 2021). This reminds us that we should pay attention to students’ Internet addiction during the COVID-19 pandemic. Numerous studies have shown that the negative impact of lockdowns caused by the COVID-19 pandemic on students with low self-esteem is significant. This means that schools and society should provide more support and attention to students with low self-esteem (Testoni et al., 2021; Vall-Roqué et al., 2021).

In addition to the research hotspots in this field, some neglected issues also need to be highlighted. First, suicide is the most serious consequence of negative mental health, but it has not been a research focus. Suicide and self-harm as a result of the direct and indirect effects of the COVID-19 pandemic is a major public health problem, and the social, psychological, and economic consequences of COVID-19 have the potential to impact global suicide rates (Farooq et al., 2021). The suicide rate of students has always been a serious problem, and the negative psychological state, financial burden and low self-esteem of students can lead to the increase of suicide rate (Toprak et al., 2011; Zhang et al., 2017; Duffy et al., 2019; Otsuka and Anamizu, 2019). Although there are relevant studies on the relationship between COVID-19 and student suicide rates and tendencies, this research direction should receive more attention (Hou et al., 2020; Fuse-Nagase et al., 2021; Marutani et al., 2021). Also, it is important to note that since suicides may be reported as unexpected events in statistics, this phenomenon may be underestimated (Pompili et al., 2012). Additionally, in addition to alcohol and Internet addiction, students’ marijuana, tobacco, and drug abuse issues during COVID-19 also require attention (Chaffee et al., 2021; Farhoudian et al., 2021).

From the results of this study, we found that the sources and influencing factors of students’ mental health problems during COVID-19 are extremely complex, which means that a single-approach solution may not be satisfactory. We need multidisciplinary support and multi-institutional collaboration. Such as the multidisciplinary consortium established by Odone et al. with expertise in the fields of economics, social sciences, epidemiology, public health and clinical medicine (Odone et al., 2020). Multidisciplinary competencies along with appropriate funding and access to rich data sources will go a long way toward achieving this goal. A holistic view of the physical, psychological, family and society is crucial to the search for solutions.

In addition, based on our results, it is also important to focus on specific groups of students, including students with addictive habits, students suffering from illness, and students with financial difficulties. It is reasonable to assume that the COVID-19 pandemic exacerbated the plight of these students and significantly damage their mental health. In terms of addictive behaviors, numerous articles have demonstrated the strong relationship between social media addiction, alcohol addiction, drug addiction, smartphone addiction, and other addictive behaviors with COVID-19 and mental health (Sujarwoto et al., 2021; Hu et al., 2022; Lardier et al., 2022; Yehudai et al., 2022). In 2020, a study focusing on children with attention deficit hyperactivity disorder (ADHD) during the COVID-19 pandemic demonstrated that the COVID-19 pandemic further exacerbated these children’s mental health and worsened their behavioral problems (Zhang et al., 2020). There are not many similar studies, but research into the psychological profile of students with disease burden may be a future research trend in this field. There have been many reports on the financial stress of students (Jones et al., 2021; Myhr et al., 2021). However, due to the various factors that cause personal economic stress, such as family, society, region, economy, etc., this direction is still worthy of further research.

Our study has two major limitations. First, we only searched literature data on WoSCC and included only English literature, which may lead to selection bias. Second, authors with the same name and the diversity of keyword expressions can lead to bias.

## Conclusion

Since the COVID-19 pandemic, the number of publications related to student mental health has increased dramatically. China and the United States lead in the quantity and quality of publications in this field. This article is the first bibliometric analysis based on the mental health of students during COVID-19 to study the research trends and hot spots. From the detected clusters of citations, students may continue to face difficulties with financial burden, anxiety and poor sleep. Student mental health may be influenced by other factors such as internet and alcohol dependence, which requires further exploration. With the assistance of information visualization, we identified the research hotspots in this field continue to revolve around emotional anxiety and unhealthy behaviors. Further study is needed to investigate the relationship of mental issue and various stressors and the interventions that effectively regulate psychological stress.

## Data availability statement

The original contributions presented in the study are included in the article/Supplementary material, further inquiries can be directed to the corresponding authors.

## Author contributions

QZ and MZ designed this study. QZ, MZ, YY, and QC reviewed and revised the manuscript. QC drafted the original manuscript. YY collected data, performed preliminary data analysis, and completed figures and tables. All authors approved the final manuscript submission and agreed to be responsible for all aspects of the work.

## Funding

This study was supported by grants from the National Natural Science Foundation of China (81700658 and 82270795) and the Hunan Provincial Natural Science Foundation (2020JJ3058 and 2020JJ4864).

## Conflict of interest

The authors declare that the research was conducted in the absence of any commercial or financial relationships that could be construed as a potential conflict of interest.

## Publisher’s note

All claims expressed in this article are solely those of the authors and do not necessarily represent those of their affiliated organizations, or those of the publisher, the editors and the reviewers. Any product that may be evaluated in this article, or claim that may be made by its manufacturer, is not guaranteed or endorsed by the publisher.

## Footnotes

^1^ https://www.bibliometrix.org/home/
